# The effects of body region, season and external arsenic application on hair cortisol concentration

**DOI:** 10.1093/conphys/coy037

**Published:** 2018-07-10

**Authors:** Madison Acker, Gabriela Mastromonaco, Albrecht I Schulte-Hostedde

**Affiliations:** 1 Department of Biology, Laurentian University, 935 Ramsey Lake Rd., Sudbury, Ontario, Canada; 2 Reproductive Physiology, Toronto Zoo, 361A Old Finch Avenue, Toronto, Ontario, Canada

**Keywords:** American mink, captive population, museum collection, Vancouver Island Marmot

## Abstract

Hair cortisol analysis has been used to quantify hormone levels in circulation in several mammal species. Hair remains stable for decades or centuries, allowing researchers to use archived hair samples to investigate hormone levels that span long time periods. However, several studies have found that intra-individual variability, driven by the body region from which a sample is derived, confounds measurements of systemic glucocorticoid hormone concentrations. In addition, the external application of chemical agents to hair can remove or concentrate molecules of interest. These may preclude the use of samples that have been collected opportunistically and/or those that have been housed in museum collections. Using a captive population of Vancouver Island marmots (*Marmota vancouverensis*), we found a strong effect of body region on the concentration of cortisol within hair, as well as an effect of season. Using a collection of American mink (*Neovison vison*) pelts, we found that application of the preservative arsenic in the form of a soap does not cause a significant decrease in cortisol. The marmot results suggest that intra-individual variability is not stable through time. The reason for these seasonal effects is not clear and further study is necessary. Researchers using samples from an unknown body region should exercise caution in interpreting their results. The mink results suggest that samples held in museum collections can be used to quantify cortisol, even when arsenic preservation is suspected.

## Teaser

Stress hormone (e.g. cortisol) levels can be assessed from hair, but the deposition of these hormones in hair may be dependent on the body region, and chemical treatments. Hair cortisol depended on body region. Arsenic soap, a common treatment for museum pelts, did not affect hair cortisol concentration.

## Introduction

Hair can be used as a substrate with which to quantify molecules found in the circulatory system of mammals, including hormones (Yang *et al.*, 1998; Koren *et al.*, 2002), metals (Sobańska, 2005; Bocharova *et al.*, 2013) and xenobiotics (Baumgartner *et al.*, 1989; D’Havé *et al.*, 2005). The glucocorticoid (GC) stress hormones cortisol and corticosterone can be found in hair samples and have been quantified in captive (Dettmer *et al.*, 2012; Malcolm *et al.*, 2013; Carlitz *et al.*, 2015), free-ranging (Koren *et al.*, 2008; Cattet *et al.*, 2014) and domesticated (Bennett and Hayssen, 2010; Finkler and Terkel, 2010; González-de-la-Vara *et al.*, 2011) mammals; these studies have shown correlations between hair cortisol and traits such as reproductive and social status, health and body condition and abnormal behaviours (e.g. self-injurious behaviour). Recently, several validation studies have shown that a single adrenocorticotrophic hormone (ACTH) injection does not lead to increased hair cortisol in captive ungulates (*Rangifer tarandus granti* and *R. tarandus tarandus*, (Ashley *et al.*, 2011)) but that repeated ACTH challenge does lead to increased hair cortisol levels in captive Canada lynx (*Lynx canadensis*, (Terwissen *et al.*, 2013)) and free-ranging eastern chipmunks (*Tamias striatus*, (Mastromonaco *et al.*, 2014)). Similarly, Yu *et al.* (2015) found increased levels of corticosterone in the hair of mice subjected to a social defeat paradigm, an experimental design intended to induce chronic stress. Taken together, these findings suggest that GC levels in hair accurately reflect long-term changes in HPA activity and that these changes are related to systemic GC levels.

The use of hair analysis to quantify systemic exposure is advantageous for two reasons. First, because hair grows over the course of weeks or months, hair analysis provides an opportunity to measure physiological changes on this same time scale (Kirschbaum *et al.*, 2009; Noël *et al.*, 2015). For instance, with hair analysis, it is possible to detect chronic exposure to toxins (D’Havé *et al.*, 2005; Boumba *et al.*, 2006) and to detect hormonal shifts associated with disease or pregnancy (Kirschbaum *et al.*, 2009; Thomson *et al.*, 2010; Wester and van Rossum, 2015). It can otherwise be difficult to measure these phenomena using substrates such as blood or saliva because measurements made using these substrates are sensitive to the circadian rhythms of metabolism (Price *et al.*, 1983; van Cauter *et al.*, 1996) and repeated point estimates are necessary to establish a pattern of exposure. Second, using hair samples it is possible to make a retrospective estimate of an individual’s exposure to extrinsic contaminants or GC hormones, as hair analysis has yielded biologically valid concentrations of metals (Lewin *et al.*, 1982; Bocharova *et al.*, 2013) and GCs (Macbeth *et al.*, 2010; Webb *et al.*, 2010) in hair samples that are several decades or centuries old.

Despite these benefits, hair as a substrate has several drawbacks that must be addressed. Hair cortisol concentration is known to vary within an individual depending on the body region from which the sample is drawn (Macbeth *et al.*, 2010; Ashley *et al.*, 2011; Carlitz *et al.*, 2015). This effect appears to be species-specific, therefore a test for the relationship between body region and hair cortisol concentration should be undertaken before interpreting cortisol values drawn from hairs that have been collected opportunistically. It is not clear why hair cortisol varies by body region. Carlitz *et al.* (2015) investigated the possibility that it may be due to blood flow at the skin, which varies at different parts of the body and therefore delivers variable amounts of cortisol to the hair follicle. They found some evidence for this in a study of chimpanzees, *Pan troglodytes*, but were not able to explain hair cortisol concentration in the shoulder using temperature alone, as they could with other body regions. They suggest that cortisol may be ‘washed out’ of hair by water, ultraviolet radiation or other external factors. When human hair was subjected to a hot water bath (40–80°C), a shampoo treatment or 9 h of ultraviolet irradiation *in vitro*, cortisol concentration in the hair decreased (Li *et al.*, 2012). However, hair samples derived from many free-living wild animals are not likely to be subjected to such conditions. Moreover, Macbeth *et al.* (2010) did not find a weathering or washout effect in grizzly bear (*Ursos arctos*) hair that was left in experimental hair snares exposed to the elements for up to 18 days. To our knowledge, despite several studies on changing hair cortisol concentration along the hair shaft, no published studies have measured hair cortisol concentration at different body regions through time to determine whether relative cortisol concentrations at each location are static within an individual. We measured hair cortisol concentration at five body regions during two seasons to establish the pattern of intra-individual variation in Vancouver Island marmots (*Marmota vancouverensis*). We expected body regions to be significantly different, but that this would not vary by season (Carlitz *et al.*, 2015).

Another challenge to interpreting hair cortisol concentrations is that hair can be altered by external application of chemicals. It is well known in the forensic science community that results of human hair analysis can be distorted by cosmetics. A shampoo and a commercial dye, for instance, were both shown to remove opiate drug metabolites from human scalp hair (Jurado *et al.*, 1997). By contrast, hair moisturizing or conditioning products may cause hair to absorb and retain xenobiotics from the environment (Kidwell *et al.*, 2015). In both cases, external application of chemical adulterants leads to unreliable results regarding systemic exposure. Although most non-human animals do not have their hair treated with cosmetic chemicals, specimens that are kept in natural history collections may be subjected to chemical treatment and it is unclear how these might affect the concentrations of hormones or toxins within the hair (Bocharova *et al.*, 2013). Fumigants and poisons were used throughout the 1800s and early 1900s by museums to preserve their natural history collections, including study skins. Among these are arsenic, mercury, tobacco and camphor which may have been used as recently as the 1960s (Goldberg, 1996). It may not always be clear which specimens have received which treatment and not all museums have the resources to test their collections in full (e.g. The Victoria and Albert Museum in London (Cullen, 2008)). It is reasonable to assume that all taxidermy specimens collected before 1960 may have been treated at some point with a toxic substance such as arsenic (Marte *et al.*, 2006). Fortunately, many museum collectors and preparators left behind detailed instructions on the preparation of study skins for museums. As such, it is possible to recreate the treatments outlined in these documents to conduct an experimental test for the effects of preservatives on the detection of hormones or other molecules within hair samples.

In 1965, R. M. Andersen at the National Museum of Canada (now known as the Canadian Museum of Nature) published the fourth edition of Methods of Collecting and Preserving Vertebrate Animals. Therein he describes the preparation and application of ‘arsenical soap’ as a treatment for mammal pelts. Arsenical soap serves as both a cleansing agent and preservative by depositing arsenic compounds onto hair to kill arthropod pests (Marte *et al.*, 2006). Arsenic is toxic in part because it has a strong binding affinity for sulphur (Platanius, 2009) and will bind to and denature proteins rich in cysteine; keratin is one such protein (Shen *et al.*, 2013). Therefore, the washout of compounds found within hair by arsenical soap may be exacerbated by damage to the hair shaft as a result of arsenic interacting with keratin. In order to understand how some of these chemicals may alter hormone levels in fur, we conducted an experiment designed to recreate the arsenical soap treatment outlined by Andersen. We measured cortisol levels in fur before and after pelts were treated with arsenical soap, prepared and applied to the skin as per Andersen (1965), with consideration of the writings of Schmidt (1824). We expected treatment with soap to have a significant washout effect on hair cortisol concentration.

Hair cortisol analysis is an efficient way to monitor and investigate the hypothalamic-pituitary-adrenal axis with the aim of understanding how individuals balance their energy budgets (Kersey and Dehnhard, 2014). As this has implications for reproduction and survival, HPA axis activity is often of interest to conservation biologists (Kersey and Dehnhard, 2014). A great deal of data likely exists in hair samples that have already been collected and stored for other purposes (e.g. natural history collections) and more could be collected easily at little cost using this non-invasive sampling technique. It is therefore important to understand what factors affect the concentation of cortisol in hair (Carlitz *et al.*, 2015). Once this has been established, hair cortisol analysis may be an appropriate technique for use in the conservation of many mammal species, the majority of which are, by definition, covered in hair and more than 20% of which are currently vulnerable to extincton (IUCN, 2017).

## Methods

### Experiment 1: body region and season

#### Study animals

A population of Vancouver Island marmots (*Marmota vancouverensis*), housed at the Toronto Zoo, was used to determine if there is an effect of body region or season on hair cortisol concentration. In Vancouver Island marmots, moulting occurs in July, beginning with adult males and non-reproductive females, followed by young adults, yearlings and finally females that have weaned a litter and their young (Naughton, 2014). We collected hair samples from five different body regions (hindlimb, chest, forelimb, rump and back) in late March and late August, to compare hair cortisol concentrations after a period of hibernation and concentrations immediately following the moult in active marmots. Vancouver Island marmots were housed in a breeding facility that is not accessible to the public. With the exception of one yearling marmot, all marmots were housed in breeding pairs. Marmots hibernate from October until April and emerge from their burrows in May. Hibernating marmots were sampled in late March without the use of anaesthetic. A second sample was taken in late August when marmots were anaesthetized for routine veterinary care using isofluorane gas. This procedure was approved by the Toronto Zoo’s Animal Care and Research Committee (REF No. 2016-03-01).

#### Sample collection

A 2 cm × 2 cm patch of fur was shaved from the left hindlimb, back, rump, chest and right forelimb of each animal using clippers to cut as close to the skin as possible. Clippers were cleaned using 70% isopropanol and compressed air between samples. Samples were collected in plain white envelopes and stored at room temperature until use.

#### Hair cortisol analysis

The hair cortisol extraction protocol was adapted from Mastromonaco *et al.* (2014) and Majchrzak *et al.* (2015). Briefly, the hair was cut into segments <0.5 cm in length and weighed in glass scintillation vials. To wash each sample, 0.75 ml of 100% methanol was added and the sample was vortexed for 10 s. The wash liquid was pipetted off and the vial was left open for 5 min for remaining methanol to evaporate. Cortisol was extracted from washed samples using 1 ml 100% methanol per 0.005 g of hair. Samples were extracted during a 24-h incubation on an orbital shaker (MBI Orbital Shaker; Montreal Biotechnologies, Montreal, Canada). Following incubation, samples were spun in a centrifuge at 3500 rpm for 10 min. Hair was discarded and 1500 μl of extract was pipetted into a new vial and dried in a fume hood under constant air flow for 24–48 h.

Dried extracts were reconstituted by adding 150 μl of phosphate buffer (0.1 mM sodium phosphate, pH 7.0, 9 g of NaCl and 1 g of bovine serum albumin per liter) and vortexing for 10 s. Cortisol values were determined using an EIA that has been previously described by Terwissen *et al.* (2013) with modifications made by Majchrzak *et al.* (2015). Cortisol antiserum (R4972, C. Munro, University of California, Davis, USA) was diluted 1:12 000 in coating buffer (50 mM bicarbonate buffer, pH 9.6) and horseradish peroxidase anti-immunoglobulin (C. Munro, University of California, Davis, USA) was diluted 1:34 000 in phosphate buffer. Molecules that are cross-reactive to the cortisol antibody are: cortisol (100%), prednisolone (9.9%), prednisone (6.3%), cortisone (5%), corticosterone (0.7%), 21-deoxycortisone (0.5%), deoxycorticosterone (0.3%). Inter-assay CVs were calculated by running external controls at 25% and 65% binding in duplicate on each plate. The CV for high control (25% binding) was 9.2% and for low control (65% binding) was 5.6%. Along with monitoring the CV of each duplicate, intra-assay CVs were further evaluated by loading a pooled faecal extract diluted to 50% binding repeatedly across the plate. For this assay, the intra-assay CV was 3.6%. Cortisol standards used were 0.078–20 ng/ml = 78–20 000 pg/ml (Steraloids Inc., Newport, USA; cat # Sigma H-0135).

Microtitre plates were incubated overnight at 4°C with 50 μl per well of cortisol antiserum in coating buffer. Plates were washed with a 0.02% Tween 20 solution using a microplate washer (BioTek Instruments, Winooski, USA) and 50 μl of reconstituted hair extract, standard or control were pipetted into the plate in duplicate. This was followed immediately by 50 μl per well of horseradish peroxidase in phosphate buffer. Plates were further incubated for 2 h at room temperature and washed again using a 0.02% Tween 20 solution. Finally, 100 μl of substrate solution was added to each well (50 mM citrate, 1.6 mM hydrogen peroxide, 0.4 mM 2,2′-azino-di-(3-ethylbenzthiazoline sulphonic acid), ph 4.0) and plates were incubated a final time for 30–45 min at room temperature. Absorbance at 405 nm was measured using a spectrophometer (Dynex Technologies, Chantilly, USA).

#### Statistical analyses

All data were transformed with the natural logarithm to meet assumptions of normality. A mixed ANOVA was used to determine the effect of body region and season on hair cortisol concentration. Tukey’s HSD was used to determine main effects of body region or season on hair cortisol concentration, and any changes in body region with time. The intraclass correlation coefficient (ICC) was calculated to determine the repeatability of cortisol concentrations within individuals. Per McGraw and Wong (1996), the ICC was designated as C,1 (two-way mixed effects model) for the calculation of a 95% confidence interval. The null hypothesis was that the true ICC of a marmot population is 0. The statistical test and transformation were performed using R statistical software (R Core Team, 2015).

### Experiment 2: museum treatment

#### Pelt preparation

To test the effects of arsenical soap, twelve American mink (*Neovison vison*) were collected from a fur farm in Southern Ontario, Canada. They were presumed dead from natural causes and were found by farm personnel in their cages on the mornings of 5 and 6 July 2016. All remains were stored at −20°C and were gradually brought to 4°C before being skinned and scraped to remove excess tissue. Pelts were hung to dry fur side out in a fume hood on a wooden board, a wire rack or a plastic rack. Drying times varied from 24 to 48 h.

#### Arsenical soap preparation

Arsenical soap was prepared following the recipe used by the field naturalists with the Canadian Museum of Nature (Andersen, 1965). In a fume hood, 113.5 g of a white laundry soap bar (sodium tallowate, sodium cocoate, sodium palm kernelate, glycerine; The Soap Works, Toronto, Canada) was melted in 75 ml of distilled water over low heat, stirred regularly. Once soap had melted to a thick liquid, 21.25 g of potassium bicarbonate (KHCO_3_, Cas Brewhouse, Sudbury, Canada), 113 g of white arsenic (As_2_O_3_, Fisher Scientific, Hampton, USA), 20 ml of pure essential camphor oil (*Cinnamomum camphora*; Puresource Inc., Guelph, Canada) and 10 ml of 95% ethanol were added. The solution was stirred until it reached uniform consistency, poured into a clean, glass Mason jar (Bernardin, Toronto, Canada) and left overnight to re-solidify.

#### Sampling and treatment of skins

Arsenic-free samples were taken from the left rump of each skin and arsenic-treated samples were taken from the right rump (as in Ashley *et al.* 2011). A small, 2 cm × 2 cm patch of fur was shaved using a beard trimmer (Maxtrim Model GMT17SDMC; Conair, East Windsor, USA) to cut as close to the skin as possible. Each pelt was washed using arsenical soap (Andersen, 1965). Specifically, 0.01 g of arsenical soap and 1.5 ml of distilled water were lathered using a paint brush. The lather was applied to the skin, first on the leather, then on the fur (Schmidt, 1824). The lather was worked into the skin using the brush for 10 s on each side and the skin was then rinsed liberally with distilled water. All skins were left to dry overnight in the fume hood. Once dry, each pelt was sampled a second time to obtain an arsenic-treated sample.

#### Hair cortisol analysis

The samples were analyzed as described above.

#### Statistical analysis

All hair cortisol concentrations were transformed with the natural logarithm to meet assumptions of normality. A paired two-tailed t-test was used to compare hair cortisol concentration in mink pelts before and after arsenic treatment. The statistical test and transformation were performed using R statistical software (R Core Team, 2015).

## Results

### Experiment 1: body region and season

**Figure 1:**
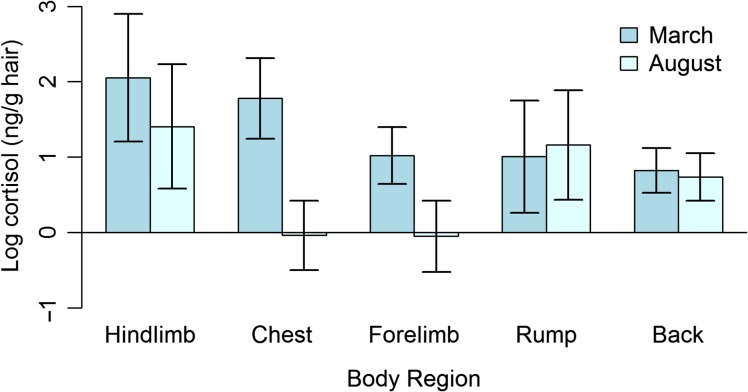
Mean hair cortisol concentration at different body regions in eight captive Vancouver Island marmots during March and August. Hair cortisol concentration is given as the log-transformed concentration of cortisol (ng) per gram of dry hair. Error bars indicate 95% confidence interval. There is a main effect of sample month (*F *= 17.57; df = 1, 63; *P* < 0.0001) and body region (*F* = 6.37; df = 4, 63; *P* = 0.00023) and a significant interaction (*F* = 4.57; df = 4, 63; *P* = 0.0026). The chest is significantly different between seasons and the left hindlimb is significantly different from the right forelimb, the rump and the back.

Eight marmots living in the Toronto Zoo were sampled in both March and August. One marmot that was sampled in March died before sampling in August. This marmot was 10 years old and in poor health so these data were omitted from analysis. The sample included data from three females and five males ranging in age from >1 year to 7 years. A mixed ANOVA showed that there was a significant main effect of body region on hair cortisol concentration (*F* = 6.37; df = 4, 63; *P* = 0.00023), a significant main effect of season (*F* = 17.57; df = 1, 63; *P* < 0.0001) and a significant interaction (*F* = 4.57; df = 4, 63; *P* = 0.0026). The left hindlimb was significantly different from the right forelimb (Tukey HSD: 1.03; *P* = 0.041), the rump (Tukey HSD: 1.045; *P* = 0.037) and the back (Tukey HSD: 1.23; *P* = 0.0076) if season was held constant. Cortisol measurements in the chest decreased significantly between March and August (Tukey HSD: 1.82; *P* < 0.01) (see Fig. 1). The ICC for marmots measured multiple times was 0.19 (95% confidence interval 0.051–0.38).

### Experiment 2: museum treatment

**Figure 2:**
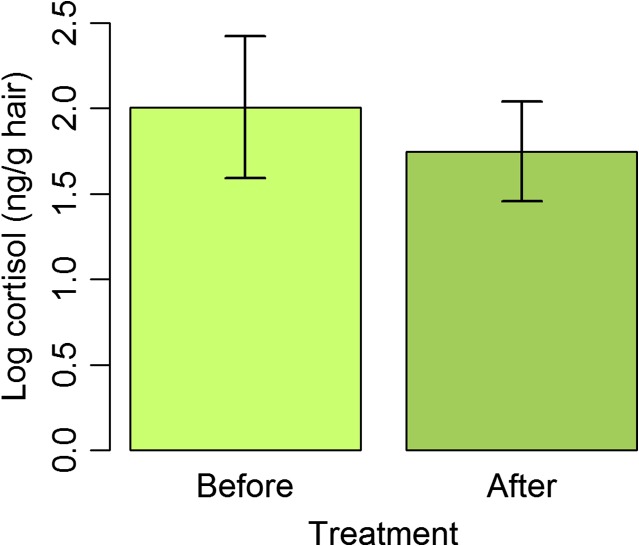
Mean hair corftisol concentration in mink pelts before and after a treatment with arsenic soap. Hair cortisol concentration is given as the log-transformed concentration of cortisol (ng) per gram of dry hair. Error bars indicate 95% confidence interval. There was no significant difference between treatments (*t* = −1.52, df = 10, *P* = 0.16).

Twelve mink pelts were sampled before and after arsenic treatment for a total of 24 paired observations. Of these, 23 measurements were above reliable detection limits. The data from the mink with the missing observation was removed from the data set, yielding a total of 22 observations from 11 mink pelts. The mean cortisol level in the mink fur before treatment was 1.97 ng/g of hair (95% confidence interval 1.51–2.42) and after treatment was 1.64 ng/g of hair (95% confidence interval 1.23–2.04). A paired *t*-test revealed that there was no significant difference in hair cortisol concentration following arsenic treatment (*t* = −1.52, df = 10, *P* = 0.16) (see Fig. 2). The estimated difference between the mean cortisol level before and after treatment with arsenic was −0.33 (95% confidence interval −0.70 to 0.046).

## Discussion

### Experiment 1: body region and season

The hair cortisol concentration in Vancouver Island marmot (*M. vancouverensis*) hair varied with body region. Regardless of season, the hair cortisol concentration in the left hindlimb was significantly higher than in the right forelimb, the rump or the back. Samples from the back were taken from the upper back between the shoulder blades. Thus, the finding that the rump and back had similar hair cortisol concentration in Vancouver Island marmots is similar to the results reported by Ashley *et al.* (2011) who found that the hair cortisol concentration in the rumps and shoulders were similar within caribou (*R. tarandus granti*) and reindeer (*R. tarandus tarandus*), and Macbeth *et al.* (2010) who found similar cortisol concentrations in rump and shoulder within grizzly bears (*U. arctos*). However, Macbeth *et al.* (2010) also found that hair on the abdomen of grizzly bears had a similar cortisol concentration to the back and rump; our results showed that hair from the ventral side of Vancouver Island marmots had a different cortisol concentration from the rump and shoulder in both the spring and the summer, but that the direction of the relationship changed. That is, in March the hair from the chest had a higher cortisol concentration, more similar to that of the hindlimb, while in August the cortisol concentration was significantly lower and was more similar to that of the forelimb. This pattern observed in the marmots from March is similar to the one observed by Carlitz *et al.* (2015) who found that hair cortisol concentration in chimpanzees (*P. troglodytes*) was higher in the chest than the back or the forelimb. Together, these findings highlight the importance of controlling for body region whenever possible (Terwissen *et al.*, 2013) and demonstrate further evidence that there is marked species-specific variation of hair cortisol concentration within individuals.

To our knowledge, our results that show two patterns of hair cortisol concentration within the same individuals sampled at different times of the year represent a novel finding. In March, hair cortisol concentration was high in the hindlimb and chest and it was lower in the forelimb, rump and back. In August, hair cortisol concentration remained relatively high in the hindlimb, but became more similar to the rump and back while the chest and the right forelimb had a lower hair cortisol concentration; these two seasons were significantly different from each other. Because the samples in March 2016 and August 2016 represent hair growth from the July 2015 and July 2016 moulting periods respectively, one could attribute that difference to different systemic cortisol levels among the marmots from one year to the next based on changes to their environment. However, the significant interaction term between body region and season suggests that some of the difference between seasons is driven by the partitioning of cortisol to different body regions from one sampling time to the next.

The ICC of 0.19 also indicates that there is little tendency for measurements within the same marmot to be similar, meaning that there is low repeatability of GC measurements in this population. In a recent meta-analysis, Schoenemann and Bonier (2018) found that the mean repeatability of GC measurements in other vertebrates (when measured using substrates such as hair and feathers) was 0.32, with a 95% confidence interval of 0.24–0.41. Thus, our results suggest below average repeatability.

These results do not lend strong support to the hypothesis that internal physiological mechanisms are solely responsible for the observed differences in hair cortisol concentration at different body regions. These results are evidence that cortisol is not deposited consistently in the hair of a given body region within an individual. If hair cortisol concentration is dependent on blood flow to a given area during hair growth, as Carlitz *et al.* (2015) proposed, there would have to be some mechanism to differentially direct blood flow away from or to the chest during a marmot’s moult. It is feasible that marmots may lose excess body heat by redirecting blood flow to the ventral skin surface during periods of intense heat. In Alpine marmots (*Marmota marmota*), laying prone on rocks or soil during the heat of the day is a thermoregulatory behaviour that helps individuals lower their body temperature (Turk and Arnold, 1988). The coat is thinner on a marmot’s ventral side and thus it is likely easier to lose heat to the environment this way. However, the Vancouver Island marmots at the Toronto Zoo are given access to climate-controlled conditions that negate the need for such behaviour. Furthermore, average temperatures in July 2016 (23.8°C, Toronto City weather station, Environment Canada) were higher than July 2015 (21.9°C, Environment Canada) so that if marmots were spending their time outside of the climate-controlled enclosures, they would have had to lose more heat in 2016 than they did in 2015 which is not consistent with higher hair cortisol in ventral samples in 2015.

We see two alternate explanations for these results. First, it is possible that different body regions are exposed to different forces affecting hair structure that cause cortisol to be washed out at different rates (Carlitz *et al.*, 2015). Macbeth *et al.* (2010) did not find evidence of cortisol loss in grizzly bear hair samples that were exposed to weather conditions that grizzly bears might encounter in the wild. Nonetheless, it is possible that friction, grooming or some other mechanical forces applied to the hair as a part of an animal’s normal behaviour could, perhaps in conjunction with weather conditions, lead to some loss of cortisol. It might be feasible to investigate this using a behavioural study. Second, it is possible that hairs in different regions have distinct properties that influence their cortisol content. Hair colour is known to influence the cortisol concentration measured within an individual (Bennett and Hayssen, 2010; González-de-la-Vara *et al.*, 2011). Marmots generally have dark brown pelage across the body with a patch of white fur at the chest (Naughton, 2014). Although effort was made to select brown hairs, it is possible that white hairs were included in the chest samples collected from these marmots and this could account for the considerable difference we found between chest measurements in March and August. However, this does not account for the variability at other body regions, where only brown fur grows.

Identifying a mechanism for these results is outside the scope of this study. Nonetheless, these results indicate that in addition to controlling for body region, it is important to select a body region wherein hair cortisol concentration accurately reflects systemic cortisol. In Vancouver Island marmots, it is clear that if one consistently sampled from the chest, one would arrive at a different conclusion regarding systemic cortisol levels than if one consistently sampled from the hindlimb. The hair cortisol concentration in the chest declined significantly between sampling periods, while the hindlimb, forelimb and back all declined slightly and the rump increased slightly. Despite this variability, the 95% confidence interval about the ICC reported here does not include 0, which we interpret as evidence that some underlying pattern is present. It is possible that such a pattern was difficult to detect owing to our small sample size. However, it is plausible that some body regions could be designated as appropriate sampling sites. Given the concordance between the difference in hair cortisol measured in the hindlimb, forelimb and back, these may be appropriate candidates for sampling areas in future marmot studies and a study designed to investigate the correlation between sampling sites within a marmot should include these regions.

These results would also benefit considerably from a biological validation in marmots. Several research teams have conducted validations in other species by stimulating the adrenal gland to produce cortisol using repeated injections of ACTH during a period of hair growth (Terwissen *et al.*, 2013; Mastromonaco *et al.*, 2014). However, these validation studies have not involved sampling from multiple body regions. Ashley *et al.* (2011) took body region variability into consideration in the design of their ACTH challenge in reindeer and caribou, however, they failed to induce a state of chronic stress in their animals by injecting them a single time with the ACTH analogue. Those who undertake future validation studies with a longer time course for injections (as in González-de-la-Vara *et al*., (2011); Terwissen *et al*., (2013)) should investigate this phenomenon more thoroughly by sampling from several body regions before and after ACTH challenge.

### Experiment 2: museum treatment

The application of arsenical soap to mammal skins as described by Andersen (1965) did not cause hair cortisol concentration to change in the short-term. This suggests that museum samples should not be discounted as valid sources data regarding historical cortisol levels, even if they are potentially contaminated with arsenic. The arsenical soap treatment represents a strong preservative and was selected for the test on this basis. It is composed not only of arsenic, but of camphor (another preservative) and soap which has been implicated in washing compounds out of human hair (Jurado *et al.*, 1997). Furthermore, this preservative is applied using the mechanical force of a paint brush to produce a lather. Our estimates of hair cortisol concentration following treatment with the arsenical soap are likely very conservative compared to estimates one might make following a preservative treatment that is less taxing on the specimen. This technique is also historically accurate. Not only was the soap prepared in the same manner as Andersen (1965) described, but the skins were also removed and dried as he suggested. This ensured that the pelts were treated in a manner that approximated the field techniques of naturalists and curators in the early 1900s, as opposed to fur trappers and hunters whose purposes were certainly different.

It remains a possibility that we have only detected the short-term effect and that long-term contact with small amounts of arsenical soap residue have an effect on hair cortisol concentrations. It would be possible to test the long-term effects of arsenic on cortisol concentration by sampling both specimens that test positive for arsenic and those that test negative for arsenic and have been in collection for many decades, but we were not able to investigate this. It is also possible that our test was under-powered and could not detect a true difference in hair cortisol concentration. There was high variability in our data, owing perhaps to the various circumstances which could have surrounded the death of each mink on the farm. Other researchers may be able to find a different source of pelts if a similar study were conducted in the future. Finally, while arsenic was one of the most common preservatives used in natural history collections, a variety of other chemicals have been used to treat study skins. Historical preservatives include alum, tobacco and benzene (Andersen, 1965), while borax is still in use in museums today (J. Miller, Royal Ontario Museum, pers. comm.). Thus there are several additional tests that should be undertaken to ensure that some other chemical treatments do not compromise the validity of natural history collections as sources of hormone data.

The world’s natural history collections are large. For instance, there are well over 1 million mammal specimens in collection in the USA (~1 078 616 specimens in the three largest natural history museums in the country) and more than 800 000 specimens in the Natural History Museum in London (Novacek and Goldberg., 2013). Although some of these specimens are skeletons, claws and other preparations that are not appropriate for hair analysis, there are nonetheless study skins among these collections. We expect that the data derived from specimens in collection could have a variety of applications. They could provide basic physiological information about a species that may be valuable if extant populations are too small to yield adequate sample sizes. They could also provide baseline hormone levels within a population that lived prior to the development of some novel threat to which current populations are susceptible (e.g. Bocharova *et al.*, 2013). Comparisons of hormone levels between historic and contemporary populations could then identify contemporary populations with hormone levels that deviate considerably from this baseline that may be populations of particular conservation interest. Other uses for historical, physiological data may be forthcoming and surely will be as techniques emerge which allow biologists to interact with museum collections in novel ways.
